# Ethical implications of neurotechnology in industry-academia partnerships: Insights from patient and research participant interviews

**DOI:** 10.1371/journal.pone.0330367

**Published:** 2025-09-02

**Authors:** Meredith V. Parsons, Maya Skolnik, Judith Mwobobia, Erin D. Solomon, James M. DuBois, Tristan McIntosh

**Affiliations:** Bioethics Research Center, Washington University in St. Louis School of Medicine, St. Louis, Missouri, United States of America; Kalinga Institute of Medical Sciences, INDIA

## Abstract

**Background:**

Neurotechnologies often advance through industry-academia (IA) partnerships and offer insight into brain and nervous system functions, bringing improved diagnosis and treatment options to patients. Both neurotechnology and IA partnerships pose ethical challenges that can impact research participation experiences, patient treatment, and health outcomes.

**Methods:**

Investigators conducted interviews with 16 patients who used neurotechnology devices in therapeutic or research settings. Interviews explored participants’ experiences using neurotechnology, perspectives on IA partnerships, preferences for neural data use and long-term care, and advice for future neurotechnology device users. Data were analyzed using inductive thematic analysis.

**Results:**

Participants were generally supportive of IA partnerships. However, they also recognized that these relationships could unduly influence research and clinical decisions. While participants appreciated the information shared with them prior to using the neurotechnology, informational gaps were still identified regarding the impact of devices on daily living, disclosure of relationships with industry, plans for data use and sharing, and plans for long-term care and upkeep of the device. Participants generally supported neural data sharing to advance research or improve patient care, although for some this depended on data sensitivity and how privacy would be protected. Participants advocated for post-trial access to experimental neurotechnologies and felt that responsibility for long-term care and device maintenance is best shared among companies, doctors, academic researchers, insurance companies, and patients themselves. Future device users were advised to self-advocate, maintain realistic expectations, and learn about a device before engaging with it.

**Conclusion:**

Given current and future capabilities of neurotechnologies and the data they generate, IA partnerships that develop and commercialize neurotechnologies require careful consideration and implementation of practices that meaningfully consider patient perspectives, needs, and safety. Such practices include bias management in the design, conduct, and reporting of neurotechnology research, neural data sharing and use, post-trial device access, and informed consent processes.

## Introduction

The field of neurotechnology is rapidly evolving, with a global market projected to exceed $28 billion by the end of 2032 [1]. In healthcare settings, these devices provide insight into brain and nervous system functions and support the diagnosis and treatment of conditions affecting the brain and spinal cord [2–4]. This is done by changing the function of the brain or spinal cord or recording and transmitting neural data. Neurotechnologies can be physically invasive (e.g., deep brain stimulation) or non-invasive (e.g., transcranial magnetic stimulation, EEG) and have been shown to improve treatment outcomes and quality of life for patients living with various conditions, including epilepsy, multiple sclerosis, chronic pain, and treatment-resistant mood disorders [5–7].

As the field advances, patient experiences using neurotechnology will have significant implications for the development pathways of these devices. Ethically, it is imperative to incorporate patient perspectives to ensure that the development and commercialization of neurotechnology genuinely address the needs and concerns of end-users [8,9]. Engaging patients in this capacity is akin to end-user testing, where their real-world experiences can provide invaluable insights that pre-clinical trials or theoretical models may overlook. Early engagement with patients can also contribute to a higher quality of long-term device rollout by identifying potential issues and areas for improvement before wide-scale implementation [10,11]. By valuing and integrating patient voices, developers can enhance treatment outcomes, improve the quality of life for users, and foster greater trust and acceptance of new technologies.

One common pathway for neurotechnology research and development is through industry-academia (IA) partnerships, or collaborations between universities and companies that aim to make scientific discoveries available for public use and patient care [12]. IA partnerships are complementary relationships that leverage the strengths of each entity while offsetting the other’s limitations. Universities are concentrated centers of multidisciplinary scientific expertise needed for foundational neurotechnology research, and academic medical centers in particular, provide patient access for discovery or development of these technologies. Companies often have the capital and dissemination capabilities essential for commercializing new treatments for patient care. The IA partnership landscape also involves a range of stakeholders in addition to industry partners and academic researchers, including university institutional officials (e.g., technology transfer, research contracts, and conflict of interest offices), healthcare providers, and patients using neurotechnology devices in clinical or research settings. Each stakeholder group brings a unique perspective in that they have different priorities, needs, and goals regarding the processes and outcomes of IA partnerships. While prior work has engaged some of these stakeholder groups about their perspectives on specific neurotechnologies, there is minimal literature on their perspectives about IA neurotechnology partnerships in particular [8,13,14]. One study surveyed industry partners and the public about perceptions of ethical issues in neurotechnology, but did not explore perceptions about ethical issues related to IA neurotechnology partnerships [15]. However, MacDuffie et al (2021) did uncover key differences between neural device companies and end-users regarding informed consent, patient privacy, and the level of confidence in industry’s ability to integrate key ethical concerns into neurotechnology development to benefit patients.

### Ethical and practical challenges in neurotechnology IA partnerships

IA partnerships involving neurotechnology present ethical and practical challenges that must be carefully addressed to maximize societal and scientific benefit while minimizing risk and harm [3,16,17]. Industry and academia are entities with inherently different and sometimes competing priorities, values, goals, assumptions, and responsibilities that shape the conduct of scientific work. While there are certainly areas of overlap in the goals of industry and academia, different pressures and motivations of each group (e.g., protecting intellectual property and meeting financial goals versus transparent sharing of scientific knowledge) can risk shaping research and commercialization decisions in ways that do not prioritize scientific integrity, rigorous research, patient well-being, or societal benefit, despite good intentions [18,19].

For instance, medical device market activity may incentivize industry to prioritize funding for large patient populations and avoid smaller patient populations, leaving smaller patient populations with fewer treatment options. In the realm of neural data, industry’s focus on safeguarding intellectual property can limit the ability of their academic research partners to share or publish data. Data sharing limitations can pose challenges for academic institutions, which traditionally favor transparent dissemination of research results, ultimately affecting the development of robust and innovative treatments. Further, academic researchers and company personnel alike may have such a strong desire for a new device to be successful that this mindset unconsciously biases their study design, interpretation of data, or reporting of research findings to the public, which risks leaving patients disappointed or unprepared to deal with the risks and side effects of a device [17,18,20]. Relationships between doctors and medical device companies may lead doctors to use a certain device more frequently without a substantial base of evidence or consideration of how other technologies might be a better fit for the individual patient. Finally, compensation models within industry and academia may introduce financial conflicts of interest at both individual and institutional levels that exacerbate these aforementioned risks [21,22].

Neurotechnologies are also unique from other medical devices as they can interpret and alter brain activity related to a person’s perception, behavior, emotion, cognition, sense of self, and memory [23]. The specialized capacities of neurotechnologies and their use raise additional ethical concerns that exacerbate the already complex ethical landscape inherent to IA partnerships. Such ethical issues include, but are not limited to, content and processes for informed consent for research emerging from an IA partnership, neural data sharing within the IA partnership, and responsibility for providing post-trial access and upkeep of neurotechnologies. For example, there is a growing focus on legislating neuro-specific data protection regulations, such as the amendment to the California Consumer Privacy Act and concerns expressed by members of congress, which emphasize the importance of regulations safeguarding neural data privacy [24,25]. Additionally, cases of companies such as Second Sight and Autonomic Technologies highlight the significant negative impact that companies going out of business or discontinuing their products can have on the long-term support for patients who rely on these technologies [26,27]. Furthermore, Starke et al. (2024) found that informed consent documents frequently lack detailed explanations regarding expectations for long-term device-access and upkeep, data use, and potential discontinuation of support for neurotechnologies.

### Present study: Patient and research participant perspectives

Neurotechnology IA partnerships can shape the design, conduct, and outcomes of research in a manner that affects the research and healthcare experiences of patients and research participants. This scope of influence brings to the fore the need to center the preferences, values, and well-being of device users when developing neurotechnologies. While research participant perspectives about IA partnerships are understudied, some available literature suggests that patients and participants recognize the role of IA partnerships in the scientific advancement of neurotechnology and its benefit to patient care [28]. This study aims to identify important factors for policymakers and clinical, academic, and industry stakeholders to consider when developing policies and practices to support responsible neurotechnology research and development. Understanding the experiences of neurotechnology users and their perceptions of key ethical challenges in IA partnerships is essential for fostering responsible innovation in the field. To address this critical need, we explored the perspectives of 16 patients who used neurotechnology devices in therapeutic or research settings to understand their interactions with neurotechnology devices and their views about IA partnerships that develop and commercialize neurotechnologies.

### Research questions

- How do neurotechnology patients perceive ethical challenges in IA partnerships?
- What expectations do neurotechnology patients have about IA partnerships?

## Materials and methods

### Recruitment and participants

This study was part of a broader National Institutes of Health (NIH) BRAIN-Initiative funded effort intended to explore stakeholder perspectives about ethical issues emerging in IA neurotechnology partnerships. Recruitment took place between March 29, 2023 and October 20, 2023. For the purposes of the present effort, we focus on one stakeholder group: patients. Participants were recruited to the study through the authors’ professional networks and using ResearchMatch, a national research participant registry supported by the NIH as part of the Clinical and Translational Sciences Awards (CTSA) Program, which included over 170,000 volunteer health research participants [29,30]. Volunteers in ResearchMatch whose health profiles included conditions in Table 1 were selected and contacted via email. With the goal of recruiting 15 participants, recruitment emails were sent to 397 volunteers who agreed to be contacted, instructing participants to review informed consent information and complete an online screening and demographic survey in Qualtrics. Of the 217 (54%) participants who completed the survey, 79 were excluded due to not meeting inclusion criteria, and 50 were excluded due to fraudulent participation activity. Fraudulent activity was determined by comparing participant survey responses (e.g., located within the US) with location metadata collected in the survey, as well as asking participants to repeat their responses to three selected survey questions at the beginning of the interview. Respondents who had metadata locations outside of the US, or failed to accurately repeat their responses to two out of three selected survey questions were deemed fraudulent. These two tests were often accompanied by other significant indicators of fraudulent behavior, such as improbable or impossible survey response patterns (e.g., a 19 year old with an invasive device to treat Alzheimer’s disease), or appearing to have a different name, age, or race from their survey when they appeared on Zoom. The study team reviewed survey responses and selected 50 individuals (excluding fraudulent responses) to invite to an interview, prioritizing a sample representing a variety of neurotechnology devices and medical conditions, of which 16 (32%) eligible participants completed.

**Table 1:** Demographic characteristics of patients and research participants (N = 16).

Response option	Frequency	Percent
**Role**		
Patient	6	38%
Research participant	1	6%
Both patient and research participant	9	56%
**Condition** ^*^		
Anxiety	2	13%
Chronic pain	5	31%
Depression	4	25%
Epilepsy	4	25%
Essential tremors	1	6%
Migraines	1	6%
Multiple sclerosis	4	25%
Pain management	3	19%
Parkinson’s disease	1	6%
PTSD	1	6%
Traumatic brain injury or concussion	1	6%
**Neurotechnology Type**		
Non-invasive neurotechnology device	8	50%
Invasive neurotechnology device	8	50%
**Age**		
20-29	1	6%
30-39	3	19%
40-49	5	31%
50-59	2	13%
60 or older	5	31%
**Sex**		
Male	6	38%
Female	9	56%
Other	1	6%
**Race** ^*^		
Asian	1	6%
Black or African American	4	25%
White	13	81%
**Ethnicity**		
Hispanic or Latino	2	13%
Not Hispanic or Latino	14	88%

* *Categories were not mutually exclusive, thus percentages total more than 100%*

Eligible participants were adults in the US who had experience with neurotechnology in therapeutic or research settings to treat a diagnosed medical condition. Participants who used neurotechnology devices in a research capacity were required to have received the neurotechnology device intervention, rather than healthy volunteers or a control group. We used purposive sampling to select participants representing an array of medical conditions and both invasive and non-invasive neurotechnology devices, reflecting a sample size adequate to reach thematic saturation [31–33]. Participants received a $60 gift card for completing a demographic survey and the interview, representing modest compensation unlikely to impose undue influence on participant responses [34,35]. Demographic characteristics of participants are provided in Table 1. This study was approved by Washington University IRB (protocol #202205141).

### Data collection

All interviewers had previous experience with qualitative research and received interview training from the lead investigator. Interviewer training included literature review, team discussion of project background and goals of the study, mock interviews, and shadowed interviews. Four interviewers conducted 1-hour semi-structured interviews over Zoom web conferencing software. Before each interview, participants provided verbal consent, and interviewers provided plain-language background information about IA partnerships via a five-minute PowerPoint presentation. This included a definition and examples of neurotechnology devices and described the purpose and mechanisms of IA partnerships. The presentation also provided examples of how these partnerships function, allowing participants to ask questions throughout the presentation and interview. Both the presentation and the interview guide were informed by prior work and reviewed for content and clarity by neurotechnology patients and the study’s stakeholder advisory board, which included neurotechnology patient advocates, clinicians, and academic researchers [3,36–39].

During the interview, participants were asked about their neurotechnology experiences, perspectives on IA partnerships, preferences about neural data use and privacy, and options for long-term care and device upkeep. They were also asked what advice they would provide to various other stakeholder groups, including other device users, researchers, and clinicians. The interviews were audio-recorded and professionally transcribed before being uploaded to Dedoose qualitative analysis software.

### Data analysis

Data analysis was guided by the Total Quality Framework and was approached using an iterative process [40,41]. Transcripts were coded using inductive thematic analysis [42,43]. Qualitative codebook development consisted of one interviewer (MS) reviewing a subset of transcripts and taking notes of emerging, recurring themes. This was followed by an iterative team discussion among the interviewers and the lead investigator (TM) about codes and code definitions. Codebook themes were grounded in the data, informed inductively from recurring ideas shared by participants, and organized under headings corresponding to the five topics covered in the interview guide.

The codebook broadly captured five domains of themes: 1) participant experiences with neurotechnology, 2) attitudes toward IA partnerships, 3) perspectives on brain data use and sharing, 4) preferences toward long-term care options, and 5) advice for future neurotechnology device users, researchers, and clinicians. Rare but significant narratives were captured under codes designated as “other,” and excerpts from these codes were reviewed by the study team in order to ensure adequate attention to uncommon narratives. Upon review of “other” codes, the study team found that the ideas expressed sufficiently related to existing domains of the codebook, and are included in the analyses presented in the results of this manuscript.

After reaching team consensus on codes and code definitions, two coders (JM, MS) applied the codebook to the transcripts. Interrater reliability tests were conducted against a third gold-standard coder (MP) after each coder completed the coding of five transcripts, and again after ten transcripts were coded. All interrater reliability tests achieved a kappa coefficient above 0.80. The study team met weekly during the coding process to discuss interrater reliability test results and resolve any questions or coding discrepancies. Coded excerpts from transcripts were reviewed by the study team and synthesized into brief code summaries containing salient themes within each code. Code frequencies were calculated to identify the most prominent codes.

## Results

### Participant characteristics

We interviewed 16 individuals who had used neurotechnology as part of their study participation or medical treatment. Participants had most often (56%) been exposed to neurotechnology devices in both patient and research participant settings. Half (50%) of participants had experience with non-invasive neurotechnology devices, including transcranial magnetic stimulation as well as EEG and MRI. Participants who were involved with invasive, surgically-implanted neurotechnology devices (50%) had experience with deep brain stimulation, vagus nerve stimulation, and implanted spinal cord stimulators. Devices were used to study and treat a range of conditions, particularly chronic pain (31%), depression (25%), epilepsy (25%), and multiple sclerosis (25%). Individuals over the age of 40 made up 75% of the sample, likely reflecting the fact that neurotechnologies are rarely a first line of treatment and often address conditions that develop or progress later in life. In terms of race and ethnicity, our sample was generally representative of the US population, with slight over-representation of Black or African American participants (25% of sample, 14% of US), and slight under-representation of Hispanic or Latino participants (13% of sample, 20% of US) [44]. Additional demographic characteristics of participants are provided in Table 1.

## 1 Neurotechnology experience

### 1.1 Participants shared a blend of positive experiences and challenges with neurotechnology

Positive experiences shared by participants (15/16) related to significant improvements in quality of life due to symptomatic relief, such as fewer seizures, better pain management, and less tingling and numbness in the limbs. Participants treated for mood disorders reported feeling more balanced and focused. Other participants also expressed relief at the technology helping them get a timely diagnosis.

*“All my symptoms have improved. My gait has improved. I never really had a tremor other than essential tremor, and my rigidity is better, so every symptom I had is better.” Participant 2, female, age 63, invasive device*

*“I have multiple sclerosis, so I get annual MRIs done of the brain. They’re a godsend in the sense that they help track lesions in the brain and activity—new activity—before becoming an actual MS attack.” Participant 4, female, age 50, non-invasive device*

When considering challenges about their experience with neurotechnology, participants (14/16) shared challenges related to the impact of the device on activities of daily living, including device maintenance, lengthy procedures, and self-consciousness about visible devices. Participants also shared challenges about access to care, such as traveling long distances for care, a device not being covered by insurance, or loss of access due to ending to study participation.

*“It’s a long procedure; it’s just long, and it’s annoying, and I don’t like to lay still for that long. Then in addition to that I always have to go to the bathroom, and I don’t even drink anything prior to it. Just laying still that long your back hurts, and then when they’re done, and you sit up you’re totally dizzy. Insurance sometimes doesn’t cover it 100 percent, so I’ve had to come out of pocket for quite a few MRIs in my lifetime.” Participant 6, female, age 42, non-invasive device*

*“The spinal cord stimulator has been needing to be charged every day and a half or so. Even though I’ve had it for a while, I still haven’t found a good rhythm. Though, I’m going to charge it every night before bed; because life happens.” Participant 13, other gender, age 32, invasive device*

Thematic analysis did not reveal substantive response differences between participants who had experience with invasive versus non-invasive neurotechnology, except when participants were asked whether they had direct contact with an industry representative while using their neurotechnology. Of the 7 participants who reported having direct contact with an industry representative, the vast majority of these participants (6/7) were treated with surgically-implanted neurotechnology devices. These interactions typically involved an industry representative providing information about a device before surgery, such as what the device would look like, how it would work, care and upkeep, and how long recovery would take. After the surgery, participants reported that the representative could program the device and help with any subsequent technical issues.

*“She gave me a little plastic VNS* [vagus nerve stimulator] *to show me what size it would be and what the weight would be, and she explained what the surgery would be like and how long I would—it would take for me to recover from the surgery. She told me about the upcoming outpatient surgeries to replace the batteries. She told me what my doctor would need when I see her. She would be able to see how many seizures I had and how good this device works. She told me a lot.” Participant 8, female, age 40, invasive device*

### 1.2 Information provided before using a device was generally sufficient, with a few exceptions

Participants (15/16) felt that doctors or researchers generally provided sufficient information before using their neurotechnology devices. Discussions with doctors and researchers typically included instructions about daily care, risks, side effects, and details about surgical and data collection procedures. Participants also reported having the opportunity to ask questions during these discussions and felt their questions were well answered.

*“I was told…that I would be the 38th person to have it done through their study, so it was experimental. I was told that insurance would not cover the cost, most likely, but that they [the researchers] would help us with the cost. I was told that at any point in time that I wanted to stop or have the device removed, that was my decision that I could do. Any kind of change of battery was gonna be covered by insurance, which was good to know. Let’s see. I was told the length of the experiment was gonna be six months, but, if I wanted to, I could opt to continue to give the doctors over at [University 1] data, which I’ve done. I wanted to be able to help others.” Participant 1, male, age 32, invasive device*

*“I was told what the process is for implantation. I was told how the technology works, but that it is not understood why it works, and that it is a hit-and-miss proposition. It was a temporary placement of leads and stimulator para-spinally which terminated outside of my body into a hip-worn device. That process was explained to me to trial the unit to see if I got any effect from it. I was told that the unit may have limited life. It may fail. Some patients, it doesn’t work at all.” Participant 14, male, age 71, invasive device*

However, participants identified a few gaps in information they wish they would have received before agreeing to use their neurotechnology devices (11/16). In their personal experiences learning about a prospective device treatment or study, participants often mentioned wanting to know more about how their collected data would be used, the probability of device failure, and what impact certain side effects might have on their health, such as unplanned weight fluctuations.

*“… I knew that wearing it was constantly transmitting data, but I’m like, I didn’t understand what the data would be used for, how it was going to be beneficial to me.” Participant 16, male, age 60, non-invasive device*

*“*[I wanted to know more about] *The percentage of* [device] *failure. I think I heard some of that about with the transcranial stimulation, there’s a one-in-something chance that it may work. There was a possibility of this not working.” Participant 4, female, age 50, non-invasive device*

## 2 Perspectives on industry-academia partnerships

### 2.1 Industry-academia partnerships have significant benefits, but biases should be managed

Participants expressed positive attitudes toward IA partnerships in 15 of 16 interviews. Many acknowledged that these partnerships can complement the limitations of both parties, bringing superior new treatments to market. For instance, participants recognized that academia might offer expertise and research capacity to test new devices, while industry may provide the resources to fund later-stage research or disseminate new treatments to healthcare providers and patients. Some participants felt that these partnerships hold particular value to students at universities, providing a chance to gain to gain hands-on experience and benefit their future careers.

*“Universities don’t have the funding to conduct studies, so they need private money to invest into the intellectual development, and then the investors wanna see return on profit. Plus there’s a manufacturing process and that goes with it that the universities aren’t equipped to handle. That’s the private industry part of it. It’s a perfect marriage.” Participant 14, male, age 71, invasive device*

*“I feel that the* [industry-academia] *relationships are good because if you’re doing it with students, they’ll get the first-hand real experience with it real-time…I feel that the students will learn a lot and continue to excel in their studies.” Participant 6, female, age 42, non-invasive device*

However, participants (9/16) also expressed some concerns about IA partnerships. Participants shared that companies could push for a specific scientific outcome in industry-sponsored research, or that financial relationships between industry and physicians could adversely influence clinical decisions. Oversight mechanisms to prevent undue influence on the design and conduct of neurotechnology research was an important assurance required by participants. Some participants also highlighted a lack of transparency in industry research and expressed concerns about the potential uses of neural data. These participants emphasized the importance of study findings being made available to the public and requested more information about the different ways companies and researchers could use their brain data.

*“I think because there has to be a lot of oversight, because I’ve heard of so many studies where companies end up pushing for a certain result that they want, and then the actual information gets lost.” Participant 13, other gender, age 32, invasive device*

*“If there is an access that you can get for overall findings for those things* [research results]*—and like I said, some organizations are better than others in making sure those things are published and they’re readily accessible. That’s the only thing that I care about, really.” Participant 16, male, age 60, non-invasive device*

### 2.2 Participants want to know about relationships between companies, researchers, and doctors

Participants (11/16) also expressed a desire to understand the nature of relationships and incentive structures between doctors, companies, and universities – even if only broadly. This included clarifying the role and obligations of doctors or surgeons within the relationship, how data are shared between parties, any incentives – financial or otherwise – exchanged, and whether there are any familial or personal ties to the neurotechnology company. One participant wanted to know how many different companies their doctor worked with for a single type of neurotechnology. They felt that working with multiple companies would allow the doctor to choose the best option for the patient.

*“I would like to know whether it is a one-on-one relationship versus the clinician working with multiple companies. Obviously, if you’re working on one-on-one, it’s a livelihood thing, especially if this is a major source of income, whereas if you’re working with multiple partners, then it’s like, okay, if you find something negative and that partner decides to drop you, then there’s other people. It seems like just diversification makes it easier to be more objective, in my opinion.” Participant 16, male, age 60, non-invasive device*

*“What the doctors or the university and the companies are providing for each other, even if it’s broad. They’re providing this data. They’re providing either this grant, or this grant’s to develop the technology. This money to develop the technology or, ‘Okay. We have a device that fits that. We’re gonna send that over to you.’ That kind of information. Also, as far as patient data, I would hope that it’s HIPAA compliant, but what of my data is being shared would be interesting to know as well.” Participant 1, male, age 32, invasive device*

## 3 Data use and privacy

### 3.1 Participants generally support responsible neural data sharing between industry and academia

Participants generally supported neural data sharing regardless of whether the data recipient was a company (13/16) or government agency (12/16). Further, most participants (11/16) shared few or no concerns with neural data sharing and use, provided that their privacy was maintained, and the data were used to advance research or improve patient care. Participants acknowledged that data use and analysis is necessary for informing treatment plans and research developing new treatments and discoveries.

*“I feel like that’s* [emotions and thought processes] *a little more personal, but at the same time, I think those are necessary things to understand because it’s just more than a physical thing. There’s also emotions and everything at play and how those impact not only healing but the way the procedure device is perceived. I think those are necessary as well, that if done properly, are still reasonable data points...I think just that either—’cause I feel like a lot of that, the who it came from or what it was for, doesn’t necessarily matter. If that’s either hidden or kept on the DL—it’s just not spread across everyone—it seems worthwhile.” Participant 12, male, age 24, invasive device*

Most participants (15/16) shared that they wanted their neural data to be used to benefit others and the progress of research. These individuals prioritized helping the scientific community and future patients with similar medical conditions by providing their neural data. Some participants recognized that their neural data might not assist current health conditions; however, they hoped it could be used in the future to help various populations.

*“As someone who participates in other types of research studies, I realize the ultimate good. Like I said, this is just for me. I’m willing to sacrifice, quote/unquote, some “privacy” if it’s gonna help* [the] *community as a whole. I know other people aren’t willing to, but I am. I just made that decision for myself.” Participant 16, male, age 60, non-invasive device*

*“Every year I have an MRI done, and I don’t mind, like we had talked about sharing the results or if it’s linked to me. I don’t care because I’d rather have people be able to study that and, hopefully, help other people and myself.” Participant 5, female, age 47, invasive device*

### 3.2 Participants have some concerns about neural data sharing and use

While participants generally supported neural data sharing and use, 7 of 16 participants expressed concerns about how neural data collected from neurotechnology devices are shared and used. First, in contrast to some participants, others shared concerns about what specific data are shared with other parties, particularly if data included information about their emotions or thought processes. Participants also wanted to know how privacy and data security would be protected by external parties receiving the data. Two individuals expressed the specific concern that if neural data are shared with external parties, biased interpretation may inflate the benefits of the device, or the data could be used maliciously or unethically and thus harm other individuals.

*“It’s like any time you have two groups partnering together; there’s always a fear that data may be interpreted differently just in order to make sure that the product goes to market or that if there’s an issue with the product that it’s swept under the rug. I don’t want to sound like some conspiracy theorist or anything like that, but that is a fear. It’s a legitimate concern. We can look at the history of corporate university partnerships and see that that has happened. It’s not as though it’s being—but for the most part, I think that most of these partnerships do provide a valuable service to the community.” Participant 16, male, age 60, non-invasive device*

*“…the only risk I can think of is patient data, the security of it. Who it’s going to be shared with, how securely it’s stored, whether people can hack it, how long it is kept. To a much lesser extent—this goes back to one of your later questions so we can come to that now— who’s going to benefit compensation wise from it? Doctors, companies?” Participant 15, female, age 38, non-invasive device*

### 3.3 Neural data de-identification is important to some participants

Half (8/16) of participants mentioned that they prefer neural data collected from neurotechnology devices to be de-identified before being shared with external parties such as governmental agencies or companies. These individuals believed de-identification could protect their confidentiality and prevent potential discrimination, especially if data contained participant information about thought processes or emotions. Two participants noted physicians treating patients with neurotechnology devices should be the only entity with access to identifiable neural data.

*“Well, it’s a company that I trust. A company that I have now. I do have concerns with that being shared in general. With the company that I have, not so much...My personal medical history and my records should remain private to me and to my physician. There is something on HIPAA that is supposed to protect the patient and if you’re sending data out to a third party that seems to violate HIPAA in my perspective unless the patient’s authorized that data to be submitted.” Participant 2, female, age 63, invasive device*

*“Optimally, I would love for it to be de-identified. Fear of some kind of persecution from that kind of data. Things have happened in history. A small number of people that have had this information or this specific procedure. It’d be kind of wild for that to happen if it’s a small number of people, but, again, who knows? Yeah. I think, if that’s the case, I would need to have signed an agreement.” Participant 1, male, age 32, invasive device*

## 4 Long-term care and health outcomes

### 4.1 Participants want long-term access to neurotechnology after participating in research

When asked whether research participants should be able to continue using a neurotechnology device after the end of a study, the vast majority (15/16) of individuals believed participants should be granted continued use of the device as long as the device benefits the participant’s medical treatment without undue risk or harm. Participants provided several reasons why continued use of neurotechnology is preferred. First, participants noted that effective devices can have a major positive impact on their quality of life. Second, participants mentioned their contribution to a study and felt that continued use of a device honored the data, time, and effort they provided – especially with surgically-implanted devices. Several participants also thought allowing a research participant to have continued use of a device after a study concludes may improve study recruitment by incentivizing potential research participant volunteers.

*“If you’re helping develop the tech, yeah, you were paid a few pennies for contributing your time, your hourly rate for working would be more than any of this research stuff, yeah. If you’re taking part and especially if you’re doing it to try to help development, that’s a part of what drives potential research subjects or patients to keep doing the study research to help develop tech and that active interest in what is being developed and how something might help them then or in the future, yeah. That’s what draws people to help a lot of this research, that would be a big motivator for not only getting more people on board, but also keeping them engaged and furthering research studies and if there’s any follow-up research, people would be more willing to do that as well.” Participant 15, female, age 38, non-invasive device*

*“Because I think, especially with a lot of these more invasive ones, the surgery is really—I know a lot of them they can just turn them off, but I think it is also just the process of going through getting the device settings correct and things. It’s—I don’t know. It feels important to be able to keep that. It’s become a big part of that treatment protocol for the person.” Participant 13, other gender, age 32, invasive device*

### 4.2 Participants want early discussions about long-term care & device maintenance

Regardless of whether a neurotechnology device was used in the context of medical treatment or research participation, 9 of 16 individuals emphasized the importance of having early discussions with healthcare providers or researchers about long-term care and maintenance of neurotechnology devices. Participants felt these conversations needed to happen during the informed consent process or otherwise before an individual begins using a neurotechnology device. Participants mentioned these discussions need to include information about device maintenance requirements, such as replacing batteries or updating software. They also wanted to understand how a device might impact daily activities or future care. For example, an implanted metal device may prohibit an individual from playing contact sports or receiving an MRI. Similarly, two participants emphasized conversations about side effects and impacts on the body, such as recovery time or scarring after surgery or unintended weight loss.

*“I’m thinking about that. I think one of the things is what happens if* [my physician] *retires? What is gonna happen, or how do I explain this to other doctors if I have to get another procedure? Say, “Hey. I have this in here.” Or* [University 1] *all of a sudden just goes away, or I move to a different city. Who can I explain this to that would understand it? Because it’s experimental.” Participant 1, male, age 32, invasive device*

*“As long as the patient has reviewed and understood and signed the informed consent, there shouldn’t be any issues with who and it should be outlined in that informed consent who’s responsible for the care after. Long-term care after the study ends. I think that should cover it. It should be addressed upfront, in other words.” Participant 2, female, age 63, invasive device*

### 4.3 Multiple parties share responsibility for long-term patient care & device maintenance

When asked who should bear responsibility for the long-term patient care and device maintenance after a study ends, participants thought the effort would be best shared across multiple stakeholders, emphasizing clear and ongoing communication between all parties involved to coordinate patient care and device support. This included calls for researchers and companies who benefit from research participant efforts to communicate with physicians to ensure that patients and research participants can use neurotechnology devices long-term (see Table 2). Participants (13/16) noted that companies involved in neurotechnology research were the appropriate party to provide device maintenance (e.g., battery replacement, software updates) and keep records of side effects experienced by those using the device. One individual stated that if risks are not explicitly discussed with the patient or research participant before using the neurotechnology, the industry should be responsible for helping alleviate the unintended side effects.

**Table 2:** Participant perspectives on parties responsible for long-term care and maintenance of neurotechnology devices (N = 16).

***Industry (13/16)*** “Long-term care. I would hope that the company would do that. They may not, but I don’t know. Be nice if they would pick up the tab for something like that…there’s always a possibility of something going wrong. In that case, I would think that that they implanted the device or whatever. They should be the ones responsible for if something does go wrong.” Participant 11, male, age 68, invasive device
***Healthcare Providers (13/16)*** “Again, somebody gets some kind of implant in their body, if they get a prosthetic, they get something, and something’s bothering them, it’s causing an issue, they go to the doctor, and the doctor helps take care of it. Right?” Participant 1, male, age 32, invasive device
***Patients (11/16)*** “Somebody’s going to tell you [the patient or participant] what the side effects or anything negative that can happen, so you already know ahead of time that there’s risks involved with it. I still think it mainly is put on the patient, but I still think, too, in both situations, the company and the doctor should both still be involved.” Participant 5, female, age 47, invasive device
***Academia (9/16)*** “In terms of research, it would have to be led by the researchers. At that level, they would have to measure it because they would have to pull in everybody to figure out what’s going on, why the unintended negative side effects, and how to resolve those…[costs of long-term care and adverse events] would come at both the researchers and the private companies.” Participant 10, male, age 60, non-invasive device
***Insurance (6/16)*** “It’s one of those things that, I guess, somebody, whether it’s Medicaid or whether their private insurance, if it’s something that they need for their health, then it should continue on. I would hope that it would. In one way or another, I guess, their insurance.” Participant 3, female, age 48, non-invasive device

*“Everyone. Me, as the research subject, the research people at the schools, the universities, and the providers of the technology, everybody needs to be involved because we’re all stakeholders in it. I would have to actively participate and explain or describing what’s going on and asking for some type of way to mitigate what’s going on as well. Everyone has to be involved in the process. There can be no hands off from anyone.” Participant 10, male, age 60, non-invasive device*

As shown in Table 2, physicians were cited by participants (13/16) as the most appropriate party to provide patient-facing care, assess ongoing benefit the device provides the patient, and monitor and communicate side effects or adverse events to the device company. Especially for experimental devices under development, 9 of 16 participants mentioned the important role of academic researchers in monitoring and addressing device side effects, as well as assisting with long-term costs and healthcare until an experimental device is fully developed. Two participants mentioned that research funding agencies such as the NIH could also allow research budgets to accommodate long-term costs of patient care and neurotechnology device maintenance for devices not covered by insurance. However, 6 of 16 participants noted that health insurance companies could assist with costs associated with long-term patient care and mitigating side effects for more established, non-experimental devices.

While participants felt that other stakeholder groups should be responsible for long-term care of patients, participants (11/16) also recognized the responsibilities of the patient in managing their own care. This included the patient’s responsibility to communicate with their physician and report any adverse events or negative side effects to their care team. Participants also felt patients were responsible for ensuring they fully understood informed consent information before agreeing to use a device, such as the maintenance requirements of the device, impact on daily living, and potential side effects. Similarly, three participants mentioned that patients need to stay informed about any changes or updates to their device for as long as their device is in use.

## 5 Advice for future patients and participants

### 5.1 Patients and research participants should learn about a device before undergoing procedures or participating in research

When asked what advice participants had for others considering treatment or research participation involving neurotechnology devices, most participants (13/16) emphasized learning as much as possible about a device from their doctor or through their own research before undergoing procedures or participating in neurotechnology research. Important information to know included the history of device development and use (e.g., stage of development), details about the procedures that have to take place in order to use the device (e.g., surgery), as well as the effectiveness, risks, and potential side effects of the device. Knowing how payments would be made for a device (e.g., insurance coverage vs out of pocket expenses) was also a key consideration mentioned. Some participants recommended involving others when learning about a device. For instance, participants suggested speaking with other patients who had previously used the device, or attending information sessions with a friend or family member – especially in cases where the patient may be looking to address a condition that involves impaired decisional capacity.

*“I think they have to be educated, they have to talk to other potential patients or people that will receive the treatment, do research, and have done—I think it all comes down to the research and talking to other providers and doctors and finding out what the best alternatives are for what it is that you’re seeking. It took a lot of research on my part to be comfortable with that. As I said, my results from that treatment are very positive, and I will continue to take on that kind of treatment.” Participant 10, male, age 60, non-invasive device*

*“*[I would advise patients and research participants] *To be educated. You must be your own best advocate with anything, with your health, your disease, whatever treatment you’re trying to get or are getting, your before and aftercare during.” Participant 7, female, age 56, non-invasive device*

### 5.2 Approach a new device with self-advocacy and realistic expectations

Ultimately, participants (13/16) felt that self-education efforts enabled patients to advocate for themselves when considering whether to use a neurotechnology device. Participants recommended cautiously approaching the prospect of using neurotechnology devices and ensuring they were comfortable with the experiment and their healthcare providers. They emphasized that clear communication with doctors is essential, as is being aware of potential side effects, to make informed decisions about their treatment. Some participants (5/13) also acknowledged the importance of having realistic expectations about a device’s capabilities, understanding that while many people may experience significant benefits, technologies are imperfect and may not work as expected or for as long as hoped, and there is no guarantee of success for everyone. Participants advised patients to maintain an open and positive mindset but be prepared for varied outcomes and manage their expectations accordingly.

*“Nothing is a guarantee. That there could be great results for a lot of people. There’s always that one person that it just may not work for. The fact that it’s—anything is kind of a chance. I mean, go into it with a really open mind but with a really positive mind. Just always have—realize that things may not always happen the same as it does for the next person, and hopefully, it’ll work, but there’s always that chance that it may not happen for that person. They just gotta keep the faith in it, but also, if it doesn’t, just don’t give up.” Participant 2, female, age 63, invasive device*

*“I would want them* [patients and research participants] *to understand that technologies are imperfect. That they will not behave as expected. They may behave as expected, but not for as long as you would like it to behave that way.” Participant 14, male, age 71, invasive device*

## Discussion

### Industry-academia partnerships

There is limited literature on patient views toward neurotechnology devices to inform IA research and clinical partnerships [28]. In our study, while participants generally recognized the merit of IA partnerships, they also had legitimate reservations regarding undue influence IA partnerships can have on processes and outcomes in research, clinical care, neural data sharing and use, and transparency of research results. These concerns, if left unaddressed, risk eroding public trust in neurotechnologies and in science when not managed proactively. Accordingly, both universities and companies may want to strategically foster public trust as neurotechnology development advances. Taking meaningful steps to cultivate public trust may even provide companies with a competitive advantage [45,46].

A strong foundation of trust can also further encourage research participation, enhance the chances of widespread public uptake of the technology, and increase a company’s market share [3]. Upholding ethical obligations, particularly as they relate to informed consent, are an important mechanism for cultivating public trust [47]. Strategies such as disclosure of financial relationships between companies and researchers or healthcare providers, providing descriptions of the oversight systems in place to prevent undue influence, as well as transparent and accessible (i.e., plain language) reporting of neurotechnology research results and the device development process were all noted by participants as practices that would help address their concerns with bias in IA partnerships. Further, involving a diverse and representative sample of end-users in the development lifecycle of novel neurotechnologies can center design priorities around key ethical and social considerations to produce impactful devices in a manner that facilitates sustained trust [48]. Future research should continue to explore procedures to build public and patient trust in neurotechnologies, scientists, and companies. Communication approaches that provide sufficient details about IA partnerships to facilitate trust while avoiding information overload would also benefit from further study.

### Informational needs

Strong communication and education are needed to help empower patients and research participants to make thoughtful decisions about pursuing neurotechnology research or treatment. Interviewees identified four types of information for researchers, healthcare providers, and companies to include as part of informed consent or other early discussions about neurotechnology: (1) impact a device may have on the patient’s health or lifestyle; (2) relationships (financial or otherwise) between companies, researchers, and doctors as it relates to the device; (3) whether and how neural data will be protected, shared, and used; and (4) plans for long-term care and upkeep of the device, as well as the parties responsible for coordinating related patient care and device maintenance. These topics may often already be included in informed consent processes. However literature continues to report low levels of participant understanding of these issues, particularly regarding data sharing and post-trial access [49,50]. There is also variability in the extent that patients are able to sufficiently understand and apply healthcare information to make appropriate decisions about their care, including health-related risk and benefit information conveyed during the informed consent process [47,51]. Researchers, healthcare providers, and companies should not assume that patients or participants understand and appreciate consent information simply because the information was delivered [52]. Tailoring informed consent procedures to meet the needs of individual patients is especially important when involving individuals with impaired decisional capacity.

Interviewees in this study advised future neurotechnology patients and research participants to learn about a device before undergoing procedures or participating in research, and to approach a new device with self-advocacy and realistic expectations. While cultivating a sense of agency is important to navigating one’s own care, providing comprehensive, clear, and accessible information regarding neurotechnology is a professional responsibility of healthcare providers, scientists, and others in the healthcare enterprise [53,54]. However, those motivated by scientific altruism may be vulnerable to coercion, be susceptible to therapeutic misconception, or downplay personal risks, believing that their contribution will benefit others in the future. This highlights the need for ethically robust consent processes that address both potential overestimation of personal benefits and the underestimation of risks. Important information about neurotechnology and IA partnerships must be communicated in a way that is accurate and accessible to the public, and comprehension may benefit from repeated exposure to the information, assessment of an individual’s cognitive capacity to consent, and the involvement of a legally-authorized representative or patient advocate [55,56]. Regulatory bodies, research funders, and institutional review boards (IRBs) should consider mandating these consent practices, especially in trials involving greater than minimal risk or participants with impaired cognitive capacity.

**Fig 1:**
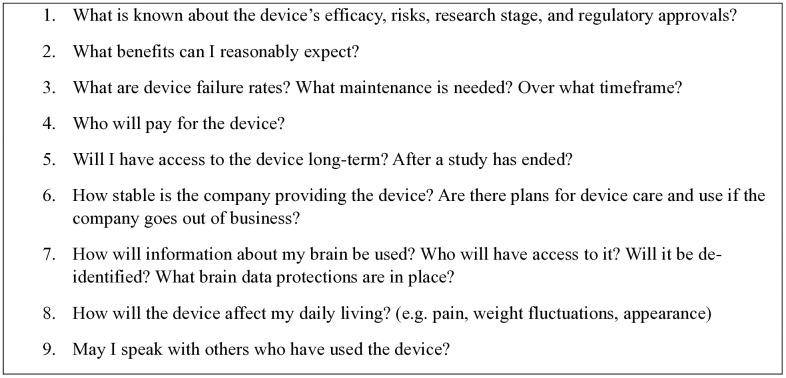
Patient-informed key questions for information gathering about neurotechnology use in clinical or research settings.

Fig 1 presents a list of questions for patients and professionals to consider when deciding whether to use neurotechnology or when developing informed consent information. These questions align with key ethical principles such as those in the Belmont report and other neuroethics-specific frameworks [57–60].This list is not exhaustive; rather, it is intended to illustrate the types of questions patients may want to ask as they are considering neurotechnology use. The informed consent process in research emerging from neurotechnology IA partnerships would benefit from further investigation as to the best ways to communicate this information. Efforts to broadly improve the informed consent process, particularly in populations with cognitive impairment, have already produced best practices and toolkits such as Consenttools.org [61–63]. Future research could also explore additional approaches and tools for supporting patient and research participant information and advocacy needs.

Patients should consider these types of questions when deciding whether to use neurotechnology. Researchers and clinical professionals should consider these types of questions when developing informed consent information.

### Neural data sharing and use

The exchange of data between universities and companies is often a necessary and beneficial practice in IA partnerships [64]. Consistent with a number of studies across multiple data types and research methodologies, neurotechnology research participation was often driven by a sense of scientific altruism, or the desire for research contributions to be used to make new discoveries, bring new treatments to market, and help future patients [49,65–67]. In this way, interviewees shared conditional approval of neural data sharing, as they recognize the benefit of reaching this goal. Although neurotechnology research participants may assume good intentions of researchers or companies using their neural data, not all participants have the subject matter expertise to fully understand current data protection practices or potential future uses of neural data [49].

Respect for privacy, assurances of data security, and protection from misuse or misinterpretation were identified as primary concerns regarding neural data sharing and use. Neuroscientists and organizations such as the United Nations Educational, Scientific and Cultural Organization (UNESCO) alike have called for neuro-specific legal protections to prevent harmful uses of neural data [68,69]. However, data de-identification and anonymization are currently loosely defined terms in biomedical research, and standardized methods for assuring patient privacy through de-identification are lacking [70]. Without professional consensus on what constitutes de-identification of neural data or a regulatory framework specifically prohibiting misuse, the good intentions of researchers and companies may not be sufficient to address research participant concerns [69,71]. This is evident in the experiences of patients from Second Sight and NeuroVista, where the companies’ closures left individuals with implanted devices that no longer functioned properly and left individuals with no available support, emphasizing the gap between good intentions and adequate patient protection [26].

Concrete strategies to mitigate bias can foster public trust and assure that practices align with patient preferences. Such strategies could include implementing robust informed consent conversations that thoroughly educate participants about the risks and uses of their data and recruiting representative sample populations to prevent skewed data interpretation. Additional strategies could include involvement of independent oversight committees to audit data handling practices and implementing effective conflict of interest management policies to help maintain objectivity in study design and reporting. Furthermore, concerns and preferences among interviewees varied substantially, demonstrating that neurotechnology device users cannot be viewed as a homogeneous group. This characterization is critical as decisions are made about how to address concerns about IA neurotechnology partnerships and neural data sharing and use.

Neural data are distinct from other forms of data in that they can contain sensitive information or carry significant implications for personal identity, cognitive function, autonomy, and mental privacy; neural data also run the risk of controversial use cases beyond medical treatment, such as human enhancement [23,72–75]. Different types of neurotechnology also produce various types of neural data, which may reveal sensitive information about an individual’s thought patterns, emotional states, memories, disease risk, or performance on certain tasks – raising critical questions for data privacy, misuse, and potential for stigmatization and discrimination [69]. Furthermore, it is unclear what analysis capabilities and use cases will be available for neural data in the future, particularly in the wake of artificial intelligence [71,76]. In some cases, de-identification may be sufficient to protect individual privacy; however, some interviewees expressed increased levels of concern with neural data sharing if data contained very personal or sensitive information [65].

Available literature regarding stakeholder perspectives on neural data sharing and use has primarily focused on researchers, leaving room for greater understanding of neurotechnology device user perspectives [77,78]. Future research should investigate what factors explain differences in comfort levels with neural data use and sharing between universities and companies, particularly across various conditions, types of neurotechnology, and data use cases. Similarly, consensus-building among stakeholders is needed to determine acceptable neural data de-identification practices and practices that protect against misuse of neural data that could harm participants, patients, or discrimination of certain groups [79,80]. Additionally, clear and comprehensive informed consent processes are crucial to ensuring that participants are aware of and agree to the potential secondary uses of their data [81]. As norms and practices related to neural data de-identification and sharing shift over time, the informed consent process must directly communicate neural data practices and procedures in place to assure privacy, security, and protection from misuse in the sharing and use of neural data [66,67,82].

### Post-trial access and long-term care

Post-trial access to neurotechnologies was perceived as honoring participant contributions to research and preserving significant improvements to research participants’ health and well-being. As observed in previous research, access to neurotechnology remained a challenge for some, highlighting the need for ongoing efforts to expand post-trial access in the field equitably [50]. Currently, many neural devices are not considered a first line of treatment, therefore, denying post-trial access proves ethically problematic when participants lack treatment alternatives [50]. This risk is exacerbated by possible loss of care continuity, an inherent gap in the US healthcare system which relies on fluctuating markets—where medical device companies may change priorities, discontinue devices, or go out of business based on changes in supply and demand—patient insurance coverage, and availability of local physicians to deliver patient care. Participants expected academics, industry, and physicians to coordinate and communicate effectively with one another and with participants regarding their neurotechnology and long-term care. While shared responsibility across these professional groups could potentially expand the range of expertise and financial resources available for long-term participant and device support, these circumstances also create conditions for a diffusion of responsibility, where “everyone’s problem becomes nobody’s responsibility ” [83,84]. Furthermore, conflicted interests among these stakeholders can shape what entities are willing to provide financial support at various points in the research and commercialization process, potentially impacting the availability of resources for long-term care and access.

Preserving participant access to beneficial neurotechnologies is a sentiment echoed in policy frameworks from both the FDA treatment investigational device exemption (IDE) regulations, the Declaration of Helsinki, and the opinion of neuroethicists and neuroscience researchers [50,85–87]. However, there is a lack of consensus on what parties hold financial, legal, and clinical responsibility for long-term participant care [55,88]. There is no consensus on how financial aspects of long-term care should be addressed and conveyed in the consent process, but other work on post-trial obligations suggest an ethical imperative to be transparent about likely long-term financial costs to the patient [50,89]. Identifying the specific responsibilities of each professional stakeholder remains an important area for future investigation. Further research, funding structures, and professional guidelines are needed to provide equitable post-trial access to neurotechnology [55].

Systems for coordinating the costs and clinical care associated with post-trial access to neurotechnology need to be synchronized early in research project design between companies, universities, healthcare providers, and potentially insurance companies [55]. In some cases, this may involve federal funding agencies allowing budget for long-term care. Contingency planning is also needed for foreseeable gaps in access to care, such as retirement or relocation of the original researcher, or an industry partner going out of business [27,90]. As the field of neurotechnology evolves, so too do cases of patients losing access to devices that generated substantial improvements to their quality of living due to company insolvency or discontinuation of devices. Cases of insolvency observed from companies such as Autonomic Technologies, Second Sight, and Nuvectra illustrate the risk to patients who were left in the aftermath to cope with painful cluster headaches, visual impairment, and chronic pain, respectively [26]. Further, systems to facilitate post-trial access need to be flexible enough to accommodate neurotechnology devices at various stages of FDA approval and testing, as well as maintain communication regarding long-term participant well-being and device maintenance between numerous siloed stakeholder groups. These plans need to be clearly communicated to the participant prior to using the neurotechnology, and potentially throughout the conduct of research studies [50]. While there are limited formal frameworks for initiating conversations about long-term care, there have been studies exploring perspectives about post-trial obligations that shed light on who should initiate these discussions [50,89,91]. We encourage professionals whose role involves performing the consent process to take the initiative in having these conversations, and to consider involving other professionals on research and clinical teams to engage in ongoing conversations about long-term care plans.

### Limitations

While our study offers unique and valuable insights about device user experiences with neurotechnology and their perspectives on IA neurotechnology partnerships in research and clinical practice, it is important to acknowledge a few limitations. The pre-interview background information provided to participants was designed to be neutrally-worded, but it is possible that some participants reported a positive attitude toward IA partnerships due to their interpretation of the material. Participants did not have an opportunity to review their transcripts, which could introduce bias, although interviewers did review the transcripts to ensure accuracy. Interviewees in this study were largely recruited using a national recruitment database; those who choose not to volunteer for research, particularly in neurotechnology patient populations, may have differing views from those included in this study. Further, our sample size (N = 16) precludes generalization of these findings or statistical comparison of differences between groups, such as individuals with invasive versus non-invasive neurotechnologies. While we reviewed subgroup differences, our subgroups were not large enough to make meaningful comparisons across demographic groups. It is noteworthy that while we reported quantities of themes, these do not necessarily equate to their importance. This presents an opportunity for future research to investigate participants of differing circumstances more robustly.

## Conclusion

To our knowledge, this study is among the first to explore the perspectives of patients and research participants regarding the use of neurotechnology devices and IA partnerships in neurotechnology development. Interviewees expressed conditional support of IA partnerships and neural data sharing, given the benefits to advancing science and medicine involving neurotechnology. Risk of bias in the interpretation and reporting of research findings, the possibility of data misuse or privacy breaches, and lack of post-trial access were primary concerns regarding neurotechnology IA partnerships identified by participants. Given the rapidly advancing capabilities of neurotechnologies and the complexity of data from neurotechnologies, it is imperative to urgently re-examine and update existing practices and policies to better support bias management, neural data sharing and use, post-trial access, and informed consent. This shift should include stakeholder co-design of future guidelines to ensure that all perspectives are adequately represented and addressed.
